# Reviewer acknowledgement 2015

**DOI:** 10.1186/s13756-016-0127-2

**Published:** 2016-08-02

**Authors:** Andreas Voss

**Affiliations:** Radboud University Nijmegen Medical Centre and CWZ, Geert Grooteplein Zuid 10, 6500 GS Nijmegen, The Netherlands

## Abstract

*Antimicrobial Resistance and Infection Control* would like to thank the following colleagues for their assistance with peer review of manuscripts for the journal in 2015.

**Sandra Alba**

Netherlands

**Jaffar Al-Tawfiq**

Saudi Arabia

**Marina Aucamp**

South Africa

**Alaa Eldin Bakr**

Egypt

**Colleen Bamford**

South Africa

**Nicholas Be**

United States of America

**Karsten Becker**

Germany

**Xavier Bertrand**

France

**John Boyce**

United States of America

**Aubonphan Buppajarntham**

Thailand

**Amit Chitnis**

United States of America

**Roy Chowdhury**

Australia

**Peter Collignon**

Australia

**Pansachee Damronglerd**

Thailand

**James Davis IV**

United States of America

**Marlieke de Kraker**

Switzerland

**Eric Decloedt**

South Africa

**Olivier Denis**

Belgium

**Ruud Deurenberg**

Netherlands

**Angel Dilip**

Tanzania

**Yohei Doi**

United States of America

**Paul Drinka**

United States of America

**Tim Eckmanns**

Germany

**Diego Faccone**

Argentina

**Alexander W. Friedrich**

Netherlands

**Christopher Fuller**

United Kingdom

**Elizabeth Garrett-Mayer**

United States of America

**Abdul Ghafur**

India

**Mario Giuffre**

Italy

**Tobias Goerge**

Germany

**Thomas Gottlieb**

Australia

**M. Lindsay Grayson**

Australia

**Sebastian Guenther**

Germany

**Revathi Gunturu**

Kenya

**Boniface Hakizimana**

South Africa

**Sonja Hansen**

Germany

**Hitoshi Honda**

Japan

**Waleria Hryniewicz**

Poland

**Nils-Olaf Hübner**

Germany

**Paul Johnson**

Australia

**Martin Kaase**

Germany

**Mamadou Kaba**

South Africa

**Nikki Kenters**

Netherlands

**Thana Khawcharoenporn**

United States of America

**Claire Kilpatrick**

United Kingdom

**Jan Kluytmans**

Netherlands

**Marjolein Kluytmans**

Netherlands

**Robin Köck**

Germany

**Sander Koëter**

Netherlands

**Tse Hsien Koh**

Singapore

**Axel Kola**

Germany

**Andrea Kwa**

Singapore

**Yew Fong Lee**

Malaysia

**Rasmus Leistner**

Germany

**Gabriel Levy Hara**

Argentina

**Moi Lin Ling**

Singapore

**Daniel Livorsi**

United States of America

**Warren Lowman**

South Africa

**David Lye**

Singapore

**Frauke Mattner**

Germany

**James Mckinnell**

United States of America

**Mary-Louise Mclaws**

Australia

**Shaheen Mehtar**

South Africa

**Leonard Mermel**

United States of America

**Barbara Middendorf**

Germany

**Katerina Mougkou**

Sweden

**Lydia Mudzikati**

Botswana

**Cathryn Murphy**

Australia

**Rahul Nandre**

United States of America

**Linus Ndegwa**

Kenya

**Lindsay Nicolle**

Canada

**Aubonphan Pattaraporncharoen**

Thailand

**Steven Pergam**

United States of America

**Trish Perl**

United States of America

**Thomas Poovelikunnel**

Ireland

**Nattapol Pruetpongpun**

Thailand

**Celine Pulcini**

France

**Thomas Riley**

Australia

**Owen Robinson**

Australia

**Jesús Rodríguez-Baño**

Spain

**Steffen Roßlenbroich**

Germany

**Joseph Rubin**

Canada

**Frieder Schaumburg**

Germany

**Sabine Schlager**

Austria

**Laura Shallcross**

United Kingdom

**Robert Skov**

Denmark

**Tara Smith**

United States of America

**Andrew Stewardson**

Australia

**Sarah Stockhausen**

Germany

**Julie Storr**

United Kingdom

**Murugan Subbiah**

United States of America

**Huang Susan**

United States of America

**Nuntra Suwantarat**

Thailand

**Rita Szabó**

Hungary

**Paul Tambyah**

Singapore

**Jean-François Timsit**

France

**Barbara Trautner**

United States of America

**Serhat Unal**

Turkey

**Anneke Van Der Zee**

Netherlands

**Elsime Visser Kift**

South Africa

**Andreas Voss**

Netherlands

**Trudy Wassenaar**

Germany

**Guido Werner**

Germany

**Wolfgang Witte**

Germany

**Thomas Wong**

Hong Kong

**Wilma Ziebuhr**

Germany

**Walter Zingg**

Switzerland

